# Rapamycin Increases Collateral Circulation in Rodent Brain after Focal Ischemia as detected by Multiple Modality Dynamic Imaging

**DOI:** 10.7150/thno.32676

**Published:** 2019-07-09

**Authors:** Jixian Wang, Xiaojie Lin, Zhihao Mu, Fanxia Shen, Linyuan Zhang, Qing Xie, Yaohui Tang, Yongting Wang, Zhijun Zhang, Guo-Yuan Yang

**Affiliations:** 1 Departments of Rehabilitation, Ruijin Hospital, School of Medicine, Shanghai Jiao Tong University, Shanghai 200025, China; 2 Med-X Research Institute and School of Biomedical Engineering, Shanghai Jiao Tong University, Shanghai 200030, China; 3 Departments of Neurology, Ruijin Hospital, School of Medicine, Shanghai Jiao Tong University, Shanghai 200025, China

**Keywords:** angiography, collateral, multiple modality imaging, synchrotron radiation, stroke

## Abstract

**Rationale**: Brain collaterals contribute to improving ischemic stroke outcomes. However, dynamic and timely investigations of collateral blood flow and collateral restoration in whole brains of living animals have rarely been reported.

**Methods**: Using multiple modalities of imaging, including synchrotron radiation angiography, laser speckle imaging, and micro-CT imaging, we dynamically explored collateral circulation throughout the whole brain in the rodent middle cerebral artery occlusion model.

**Results**: We demonstrated that compared to control animals, 4 neocollaterals gradually formed between the intra- and extra-arteries in the skull base of model animals after occlusion (*p*<0.05). Two main collaterals were critical to the supply of blood from the posterior to the middle cerebral artery territory in the deep brain (*p*<0.05). Abundant small vessel and capillary anastomoses were detected on the surface of the cortex between the posterior and middle cerebral artery and between the anterior and middle cerebral artery (*p*<0.05). Collateral perfusion occurred immediately (≈15 min) and was maintained for up to 14 days after occlusion. Further study revealed that administration of rapamycin at 15 min after MCAO dilated the existing collateral vessels and promoted collateral perfusion.

**Principal conclusions**: Our results provide evidence of collateral functional perfusion in the skull base, deep brain, and surface of the cortex. Rapamycin was capable of enlarging the diameter of collaterals, potentially extending the time window for ischemic stroke therapy.

## Introduction

Brain ischemia resulting from the occlusion of cerebral blood vessels can induce brain damage and dysfunction 1. Any strategy to restore cerebral blood flow (CBF) in the ischemic region may reduce brain damage and improve functional recovery in both humans and animals 2-4. Good collateral circulation after ischemic stroke is an independent predictor of favorable outcome regardless of treatment 5-8. Promotion of collateral circulation in either currently existing or newly formed collaterals plays a critical role in reducing ischemic brain injury and subsequently attenuating the severity of lesions 9-11. Therefore, improving our understanding of collateral morphology, increasing existing collateral blood flow perfusion, and promoting new collateral formation in brain ischemia are extremely important goals. It is also vital to develop new approaches to increase collateral blood flow perfusion and extend the therapeutic time window after ischemic stroke.

The brain circulation system begins at the internal carotid artery (ICA) and extends through the anterior cerebral artery (ACA), middle cerebral artery (MCA), and posterior cerebral artery (PCA) to supply blood flow to the entire brain. In general, each artery supports only its own territory. Previous studies have suggested that collaterals do exist between the ICA and the external carotid artery (ECA), the ACA and MCA, and the PCA and MCA 12-14. However, flow across these collaterals is very low due to little or no pressure drop, and they receive perfusion only following focal ischemia 15. When and how collateral formation occurs during cerebral ischemia is not completely understood. Because the diameter of collateral arteries often ranges between 30 and 300 µm and collateral arteries are located at the skull base, the leptomeningeal/pial membrane, and the deep brain region, current small-animal imaging techniques are insufficient to assess collateral blood flow 16. Many study data involving living animals have been acquired from the limited surface of the brain rather than from the entire brain, including the skull base and deep and surface regions of the brain 17-21. Therefore, developing a feasible approach to directly and dynamically study collaterals using a multiscale approach could greatly improve our understanding of collateral perfusion and formation.

Magnetic resonance imaging (MRI), microscopic computed tomography (micro-CT), laser speckle contrast imaging (LSCI) and two-photon laser scanning microscopy (TPLSM) are all powerful tools for detecting vascular morphology in animals. Due to their limitations in spatial resolution or light penetrability, using one device is insufficient to detect collaterals entirely and precisely in small animals 22-25. Thus, developing a novel *in vivo* imaging technique that can dynamically record collateral circulation with high spatial resolution is required, especially for preclinical small-animal studies. Recently, a novel synchrotron radiation angiography (SRA) method was developed that can dynamically monitor changes in vascular morphology in real time with high resolution (20 µm vessel diameter) during stroke and myocardial infarction 26, 27. SR angiography, micro-CT, LSCI, and TPLSM together provide very powerful support for studying morphological changes in small vessels of rodents.

Rapamycin, an inhibitor of mammalian target of rapamycin (mTOR), can restore CBF and brain vascular density (BVD) via regulating nitric oxide (NO) release in Alzheimer's disease (AD) mice 28. Inhibition of mTOR delays endothelial cell (EC) senescence *in vivo* and *in vitro* 29, 30 and improves vascular outcomes after ischemic brain injury 31, 32; mTOR inhibition also induces EC-dependent vasodilatory properties 33, 34. Rapamycin is able to rescue vascular and metabolic deficits in young APOE4 mice 35. Although mTOR signaling pathways are known to be involved in cerebral vasospasm 31 and vascular integrity 28, the role of rapamycin in relation to collaterals during cerebral ischemia is obscure. The relationship between rapamycin- induced improvement in neurological functional recovery and increased collateral circulation during ischemic stroke needs to be clarified.

Our previous study successfully showed that SR angiography is a unique tool for dynamically investigating the changes in small vessel morphology in living rodents 36. In the present study, using a multimodal imaging system, we investigated collateral perfusion, collateral formation and collateral function at different vascular diameter levels in the rodent middle cerebral artery occlusion (MCAO) model. We further investigated the role of rapamycin in the regulation of collaterals during cerebral ischemia.

## Methods

### Experimental design and methods

Experimental animal studies were performed in accordance with the Animal Research: Reporting of *in vivo* Experiments (ARRIVE) guidelines with government approval by the State Agency for Nature, Environment and Consumer Protection North Rhine-Westphalia. Procedures involving the use of laboratory animals were approved by the Institutional Animal Care and Use Committee (IACUC) of Shanghai Jiao Tong University, China. During the animal studies, regulations for the Administration of Affairs concerning experimental animals in China enacted in 1988 were followed. Adult male Sprague- Dawley rats (n=96) were used for the SR angiography and micro-CT imaging study, and adult male C57BL/6 mice (n=30) were used for the LSCI study. Single or multiple imaging procedures were performed at different time points after MCAO. We chose to use both rats and mice because rats were appropriate for SR angiography and mice were appropriate for the LSCI study.

### MCAO procedure

The rat and mouse MCAO models were previously described in detail 37. Adult male Sprague-Dawley rats (250-280 g body weight) or C57BL/6 mice (25-30 g body weight) were anesthetized with ketamine (100 mg/kg) and xylazine (10 mg/kg) intraperitoneally (i.p). Their body temperatures were maintained at 37±0.5°C during surgery by a heating pad (RWD Life Science, Shenzhen, China). A midline incision was made on the neck under an operating microscope (Leica, Wetzlar, Germany), and the left common carotid artery (CCA), ECA and ICA were isolated. A silicone-coated round top 4-0 suture was used for rats, and a 6-0 suture was used for mice (Dermalon, 1756-31, Covidien, OH); in each case, the sutures were gently inserted from the ECA stump to the ICA to induce MCAO. The suture insertion distance from the bifurcation to the ostium of the MCA was 18±0.5 mm for rats and 10.5±0.5 mm for mice. The pterygopalatine artery (PPA) was ligated at the same time using an 8-0 suture. Success of the occlusion was characterized as a 20% reduction in CBF, which was verified by laser Doppler flowmetry (Moor Instruments, Devon, England). After 90 min of occlusion, the suture was withdrawn; the CCA was restored, and blood flow returned to at least 70% of the baseline blood flow.

Both the mice and rats were treated with rapamycin once by i.p. injection immediately after MCAO. One group of mice was treated with rapamycin (10 mg/kg) in 10% DMSO, and one group of mice was treated with 10% DMSO as a control. Each group contained 6 mice in each experiment. Similarly, two groups of rats (n=6 per group) were used to study the effect of rapamycin on collateral circulation. Imaging studies were performed just after MCAO and up to 14 days after MCAO based on the different techniques.

### Synchrotron radiation angiography (SRA)

SRA was conducted using a 13 W beam line at the Shanghai Synchrotron Radiation Facility (SSRF), Shanghai, China. The imaging parameters and procedures were as described previously 38, 39. A homemade angiographic catheter, used for injecting contrast reagent, was then carefully inserted into the bifurcation of the ICA and the ECA. A 10 ml syringe was fixed onto a microinjection system consisting of a precise injection pump (Longerpump, Baoding, China). The rat was placed in the right or left lateral position on a flexible platform. The position was adjusted to ensure that the animal was horizontal on the platform and facing the X-ray path in the center of the beam 40. An X-ray energy of 33.2 keV, which is just above the iodine K-edge energy (33.17 keV) threshold, was used. Nonionic iodine contrast agent (300 mg/ml, Omnipaque, GE, CT) was injected into the ECA at 130 µl/s in a total volume of 150 µl. A charge-coupled device with a resolution of 9 µm (Photonic Science, East Sussex, England) was placed 65 cm away from the animal. Ten consecutive images were taken before contrast enhancement and were subtracted from the raw images after injection to eliminate the background structure. Images were postprocessed by MATLAB (The Math Works Ltd., Natick, MA) to perform a temporal subtraction to eliminate the bone signals. To acquire a clear general view of the rat intra- and extracranial arterial system, the images were joined and modified with Adobe Photoshop CS6 (Adobe, San Jose, CA).

### Microcomputed tomography (micro-CT) imaging determination

After SRA, the animals were placed in a supine position on the operating table and deeply anesthetized by an overdose of phenobarbital. The chest cavity was perfused, and the entire heart was exposed. Then, the right atrium was perfused, and a 20 ml dose of saline was gently injected into the left ventricle. Finally, 4% paraformaldehyde buffer was perfused to fix the tissue. The newly configured Microfil contrast medium (Flow Tech, Inc. Carver, MA) was then slowly infused through the aorta at approximately 3 ml/min. When the solution infusion was completed, the brain tissue was stored at 4°C for 2 h. The tissue was handled carefully to ensure the integrity of the circle of Willis. The brain tissue was placed vertically in a 15 ml centrifuge tube and placed in a micro-CT instrument for imaging.

The Micro-XCT-200 X-ray imaging system (Zeiss, Micro-XCT, Xradia Inc., CA) was used, and the imaging parameters were as follows: energy of 40 KV, power of 8 W, and resolution of 12.5 microns. Images were acquired with the following parameters: 8 μm isotropic voxel resolution at 30 KeV and 8,000 ms exposure time, 30 mm from the X-ray source and 17 mm from the detector. The results of the micro-CT were recorded as a Tiff format image, and a reconstruction fault map was generated using the reconstruction software. The MATLAB fault maps were converted into 8-bit images for easy reconstruction. Amira software was used for the 3D reconstruction, adjustable threshold establishment, and rendering.

### Laser Doppler flowmetry and laser speckle contrast imaging (LSCI) monitoring

Before, during, and after MCAO, a probe for laser Doppler flowmetry (Moor Instruments, Devon, UK) was placed on the mouse skull 1 mm posterior and 2 mm lateral to the bregma to measure the surface blood flow. Successful MCAO was defined as regional blood flow decreasing by more than 80% compared to the baseline, and successful reperfusion was defined as a restoration of blood flow to more than 70% of the baseline blood flow. A few animals were excluded due to not meeting this criterion, and the mortality rate after surgery was 15%.

During the imaging procedure, the mice were anesthetized, and a midline incision was made. We used an LSCI setup to acquire the speckle images for the baseline image. The LSCI system was described in previous studies 41. The exposure time was 5 ms, and the frame rate was 11 fps. The mouse's cortex was illuminated by a reshaped laser beam from a 633 nm laser diode (780 nm; 10 mW; Shanghai Forward Optoelectronics Ltd., China). Two hundred speckle images were recorded from each imaging section. Similar imaging sections were recorded at different time points based on the experimental design.

Data processing for LSCI included the following: 1) CBF estimation: a temporal laser speckle contrast analysis (tLSCA) method was utilized to calculate the contrast value (Κ) based on 80-frame stack speckle images. See Equation (1).


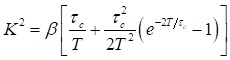
 Eq. (1)

where the correlation time *τ_c_* was assumed to be inversely proportional to the blood flow speed υ. β accounted for the loss of correlation corresponding to the ratio of the detector size to the speckle size and polarization. T was the exposure time. 2) Vessel diameter evaluation: vessel diameters were analyzed via drawing lines vertically, crossing the blood vessels of interest several times. The diameters (R, in pixels) of the vessels of interest were then determined. 3) Blood volume calculation of the vessels: the blood volume (V) was defined as the total blood flow passing through the cross section of the vessel in unit time, which is given as follows:


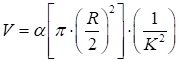


where α is an arbitrary constant.

### Infarct volume measurement

The brain infarct and edema volume were measured according to methods described in a previous study 42. Brains were rapidly removed and frozen following MCAO. The infarct volume was measured using cresyl violet staining. A series of 20 µm frozen coronal sections from the anterior commissure to the hippocampus were cut. The distance between the sections was 200 µm, and a total of 20 sections were counted. The infarct area of each section was delineated blinded to the animal's group assignment, and the infarct volume was calculated using the following formula:

V = 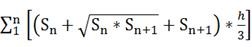


where h is the distance between two sections. Image analysis software (NIH ImageJ, Bethesda, MD) was used for infarct volume determination.

### Neurobehavioral assessments

Neurobehavioral assessments were conducted by an experimenter who was blinded to the animal's treatment conditions. A rotarod test was used to evaluate the motor and balance functions of the mice. The mice were trained to stay on an accelerating rotating cylinder for 3 days before MCAO, and the time they could stay on the rotating rod was recorded before surgery and 7 and 14 days after MCAO using methods reported previously 43. The velocity was increased slowly from 4 to 40 rpm over 2 min. For each test, every animal was tested 3 times, and the average time spent on the rod was recorded. For neurological function assessment, a modified neurological severity score (NSS) ranging from 0-14 was adopted; the assessment included raising the mouse by the tail (0-3), walking on the floor (0-3), beam balance tests (0-6), and the absence of reflexes (0-2).

### Statistical analysis

The number of collaterals, diameter of collateral arteries, infarct volume, and NSS were compared between groups by Student's unpaired t-test using SPSS software (v18.0, SPSS Inc., Chicago, IL); the values are presented as the mean±SD. For the multiple time point test, one-tailed ANOVA followed by Bonferroni's t-test was used. A probability value of less than 0.05 was considered to represent statistical significance.

## Results

### Collaterals between intra- and extracranial arteries after MCAO

**Figure 1:**
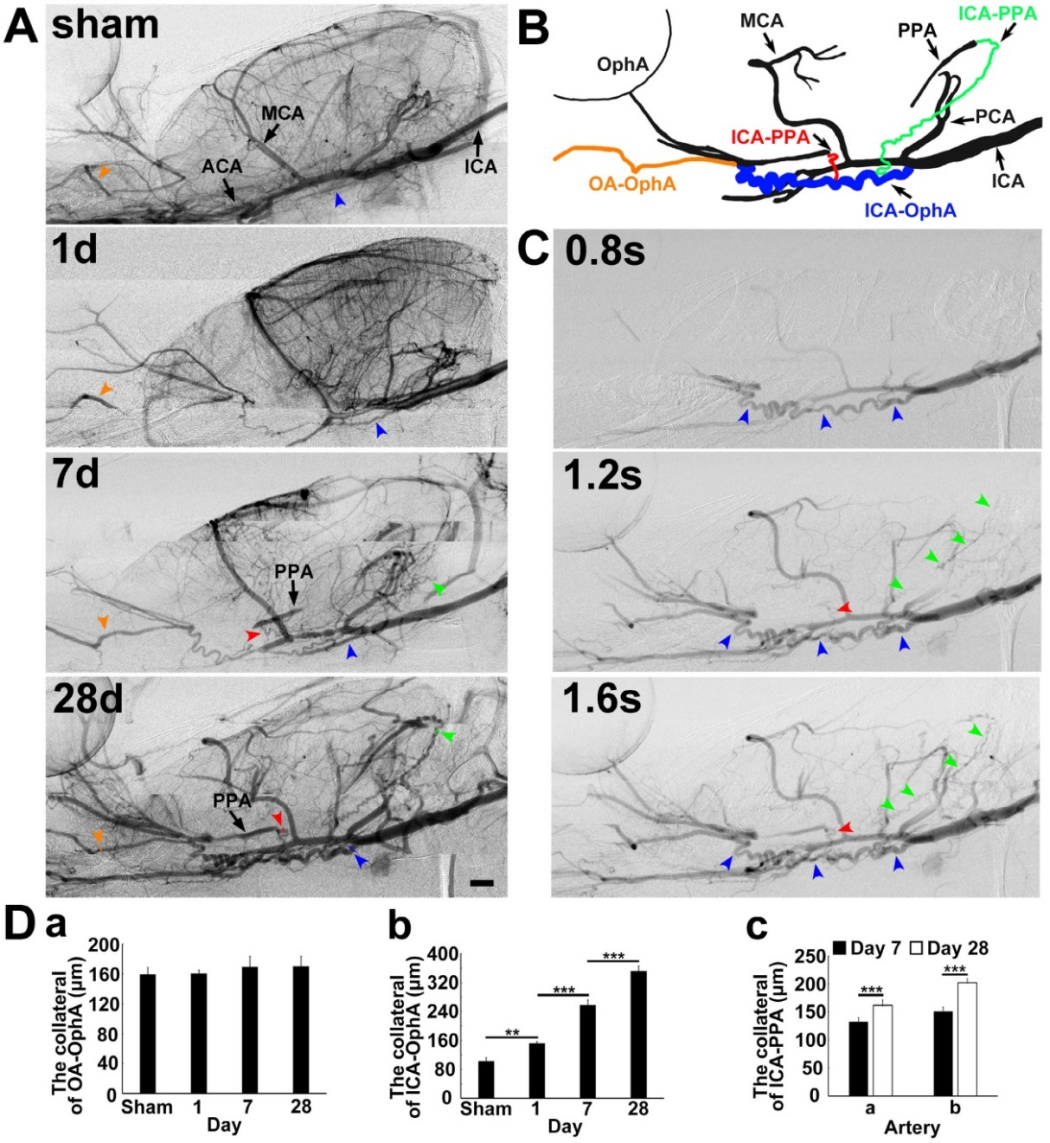
**Collateral blood flow restoration between intra- and extracranial arteries after transient MCAO**. **A**) Dynamic brain SR angiographies were achieved in living rats following 1, 7 and 28 days of MCAO with PPA ligation. **B**) Schematic diagram illustrating collateral blood flow from the internal carotid artery (ICA) to the pterygopalatine artery (PPA), the ICA to the OphA and the olfactory artery (OA) to the ophthalmic artery (OphA). The orange arrowhead indicates the collateral blood flow from the OA to the OphA, the blue arrowhead indicates the collateral blood flow from the ICA to the OphA, and the red and green arrowheads indicate the collateral blood flow from the ICA to the PPA, respectively. **C**) Dynamic SR angiographies taken at 0.8, 1.2 and 1.6 s after contrast agent injection in the rat following 28 days of MCAO with PPA ligation. The orange, blue, red and green arrowheads indicate the collateral blood flow from the OA to the OphA, the ICA to the OphA, and the ICA to the PPAs, respectively. Scale bar=1 mm. **D**) Bar graph showing the vessel diameters of the collaterals between (**a**) the OA and the OphA; (**b**) the ICA and the OphA at 1, 7, and 28 days of MCAO; and the collaterals between (**c**) the ICA and PPA a section and the ICA and PPA b section at 7 and 28 days of MCAO. n=6 per group, **p<0.01, ***p<0.001.”

To explore the collateral perfusion in the skull base between the intra- and extracranial arteries after MCAO induced by PPA ligation, we examined the skull base arteries via dynamic real-time SRA in living rats. The general parameters, including blood pressure, heart rate, body temperature, and blood gases, before, during and after MCAO were in the normal range in all experimental animals (**Supplementary Table 1**). PPA was detectable in the normal rats and undetectable immediately after PPA ligation in both the MCAO rats and the control. The blood flow in the ophthalmic artery (OphA) originated from a branch of the PPA in the normal rats and, after MCAO, partially originated from the olfactory artery (OA) as a compensatory mechanism (**Fig. 1**). We therefore named this artery between the OA and OphA the ophthalmic anastomose (OA-OphA). We found that the blood flow continuously existed in the OA-OphA from day 1 up to day 28 (**Fig. 1Da**, *p*>0.05). Blood flow perfusion in the collateral between the ICA and OphA was formed 1 day after MCAO with PPA ligation but did not exist in the normal rats; we named this collateral artery the collateral ICA-OphA. Notably, the diameter of the collateral ICA-OphA gradually increased up to day 28 (**Fig. 1Db**, *p*<0.01). These results suggest that the blood supply of OphA originated from the PPA branch but not from the ICA under normal conditions. However, the blood supply of OphA originated from the ophthalmic anastomose and the ICA after MCAO. In addition, another two collateral arteries between the ICA branch and the PPA branch (ICA-PPA) slowly formed beginning on day 7 after MCAO. Therefore, the PPA could still be partially detected even though the PPA was ligated. The diameters of these two collaterals gradually increased up to day 28 after MCAO (**Fig. 1Dc,** *p*<0.01). All of these results suggested that the intra- and extracollateral perfusion in the skull base was gradually formed after ischemic stroke, which partially maintained the blood supply of the MCA territory after MCAO with the PPA ligation.

### Collateral perfusion exists between the MCA and PCA terminals in the deep brain

**Figure 2:**
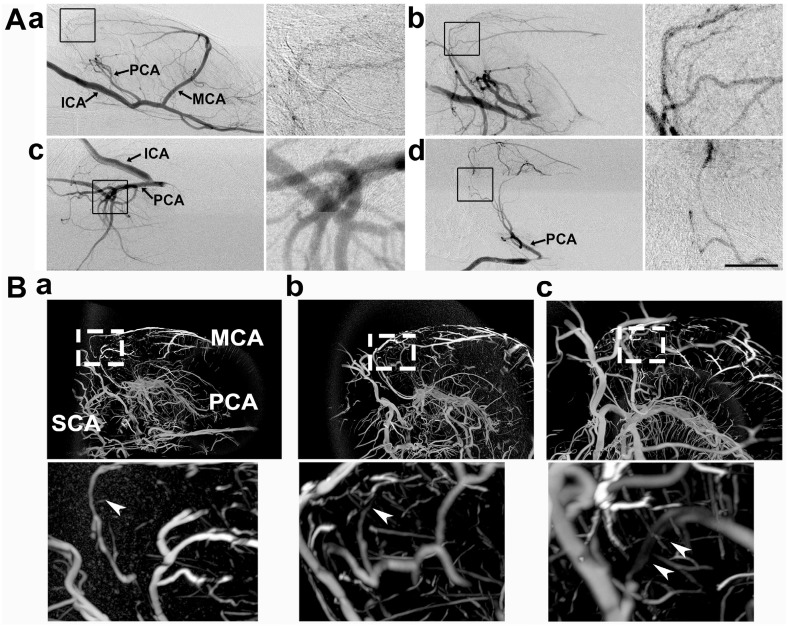
** Collateral perfusion in the rat deep brain after MCAO. A**) Dynamic sagittal SR angiographic images of collateral perfusion taken before MCAO (**a**) and 1 h after MCAO (**b**) in living rats. In **Ab**, two main collateral blood flow perfusions appeared from the PCA towards the MCA territory after MCA occlusion. SR angiographic imaging showing a (**c**) 60° dorsal position and a (**d**) 60° prone position. **Figs. a1-d1** show enlarged images of the boxed area on the left side of the four images. The arrows indicate that the collaterals perfused between the MCA and PCA in the deep brain. ICA: internal carotid artery; MCA: middle cerebral artery; PCA: posterior cerebral artery. Bar: 1 mm. n=6 per group. **B**) Micro-CT images of the collateral perfusion were rotated counterclockwise at 30 degrees (**a**), 60 degrees (**b**), and 90 degrees (**c**). Collateral perfusion from the PCA to the MCA was clearly detected. MCA: middle cerebral artery; PCA: posterior cerebral artery; SCA: superior cerebellar artery.

To detect whether collateral perfusion existed between the MCA and PCA terminals in the deep brain, we examined these terminal arteries in the deep brain during MCAO via SRA. Collateral blood flow perfusion between the MCA and PCA terminals in the deep brain was not detected in the normal rat (**Fig. 2**). The collaterals with blood flow perfusion between the MCA and PCA terminals in the deep brain were detected in 75% of rats (9/12) after 1 h of MCAO. In the other 25% (3/12), these collaterals without flow perfusion were detected even after 6 h of MCAO, suggesting that the collateral perfusion could vary among individuals. From multiple viewpoints, flow restoration from the two main collaterals was clearly extended from the PCA to the MCA (**Fig. 2A**).

To further verify the collateral between the PCA and MCA in living animals, we performed 3D micro-CT imaging via the microfilm infusion approach. The morphological results showed that the collaterals were abundant between the PCA and MCA in the deep brain after MCAO (**Fig. 2B**), which suggested that these collaterals were the most important source of blood supply in the deep brain when the lenticulostriate artery was occluded.

### Collateral perfusion was increased between the cortical MCA/ACA and MCA/PCA after MCAO

**Figure 3:**
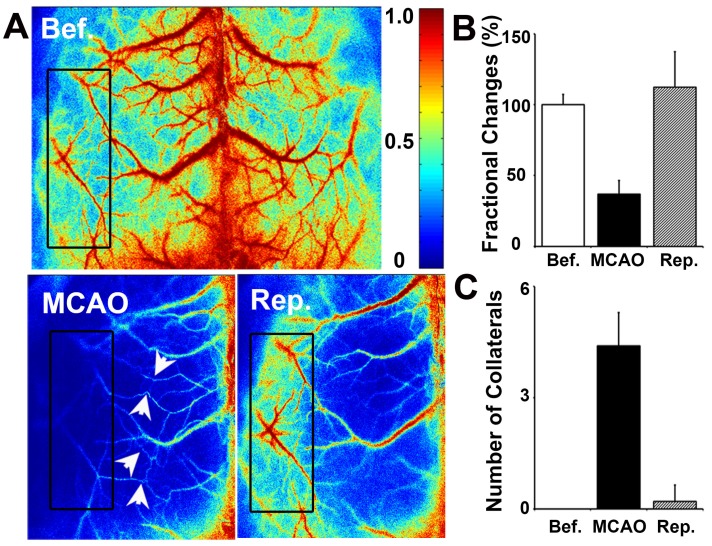
** Cortical collateral perfusion after MCAO as detected by LSCI**. **A**) Cortical blood flow was measured on the mouse skull by LSCI. The colors from blue to red represent the blood flow velocity from lower to higher. Representative images show the LSCI before MCAO (**upper**), during MCAO (**bottom left**), and after MCAO (**bottom right**) in living mice. The black box indicates the measurement of collateral circulation located on the surface of the brain in living mice. The bar graph shows the number of perfused collaterals (**B**) and the fractional changes (**C**) before and after 1 h of MCAO as well as after reperfusion. n=6 per group, *p*<0.05, before MCAO vs. after MCAO, or in MCAO vs. in reperfusion.

To determine whether collateral perfusion was increased between the cortical MCA/ACA and MCA/PCA after MCAO, we examined small cortical vessels in the living mouse skull directly via LSCI measurement. We found that small vessel collaterals existed between the MCA/ACA and MCA/PCA under normal conditions. The number of collaterals increased immediately after MCAO and almost returned to baseline after reperfusion (**Fig. 3**). Interestingly, the fractional changes were attenuated immediately after MCAO and returned to baseline after reperfusion. This result suggested that cortical collateral perfusion was a critical source of blood supply during MCAO, which could partially reduce ischemia-induced cortical injury.

### Rapamycin treatment improved neurological outcomes

**Figure 4:**
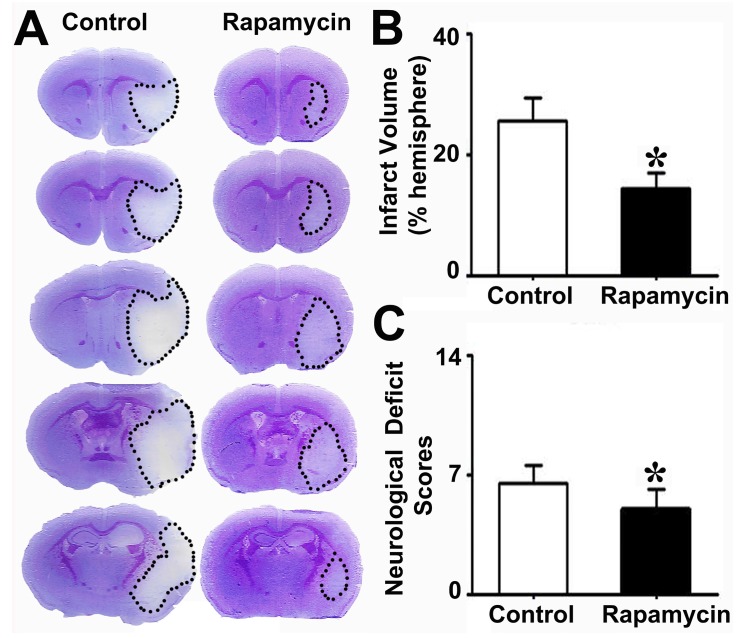
** Reduced brain infarct volume and neurological severity score in rapamycin-treated mice**. Photomicrographs showing the cresyl violet-stained brain coronal sections in the rapamycin-treated and control mice (**A**). The dotted white colored areas are the injured brain tissue. The bar graph shows the infarct volumes (**B**) and neurological severity scores (**C**) in these two groups of mice. n=6 per group, *p<0.05, rapamycin-treated mice vs. control mice.

To explore the effect of rapamycin on neuroprotection after MCAO, we injected rapamycin into mice i.p. during MCAO and then examined their infarct volume and NSS. We found that the infarct volume in the rapamycin-treated mice was smaller than that in the MCAO-alone mice. Similarly, the NSS of the rapamycin- treated mice was improved compared to that of the MCAO-alone mice (**Fig. 4**).

### Rapamycin treatment increases collateral perfusion after MCAO

**Figure 5:**
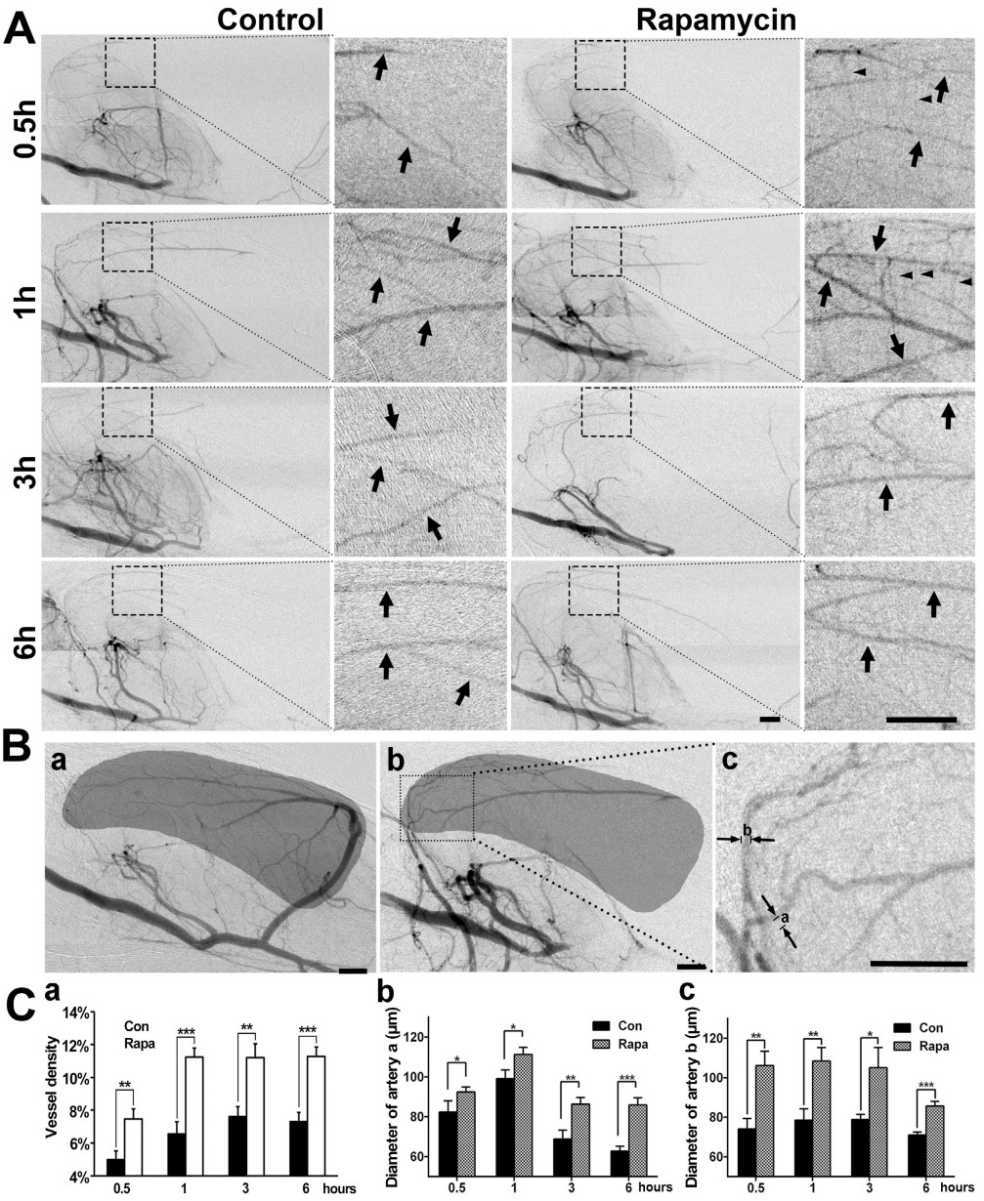
** SR angiography shows that rapamycin improves the blood supply in the MCA territory. A**) Dynamic SR angiography showing the changes in PCA blood flow and its branches at 0.5, 1, 3 and 6 h of MCAO in rapamycin-treated and MCAO-alone rats. Arrows indicate the enlarged view of PCA branches and that these branches are towards the MCA territory after MCAO. The small arrowheads indicate the cortical penetrating artery. Scale bar: 1 mm. **B**) SR angiography showing the MCA territory in normal (**a**) and MCAO rats (**b**). The shadowed areas illustrate the region of the microvessel selected for measuring microvessel density. In figure (**c**), the diameters of a and b indicate 2 main collateral arteries from the PCA towards the MCA territory during MCAO. **C**) Bar graph showing the microvessel density (**a**), the diameter of collateral a (**b**), and the diameter of collateral b (**c**) after 0.5, 1, 3 and 6 h of MCAO in rapamycin-treated and MCAO-alone rats. Scale bar: 1 mm. n=6 per group, *p<0.05, **p<0.01, ***p<0.001, rapamycin-treated vs. control rats.

To examine whether the neuroprotective effect of rapamycin was related to collateral perfusion during MCAO, we analyzed the collateral perfusion rate and the diameters of the main collaterals in the deep brain of rats (**Fig. 5**). SRA showed that the perfused collateral rate was 76.9% (10/13) in rapamycin-treated rats and 75% (9/12) in the MCAO-alone rats following 0.5, 1, 3, and 6 h of MCAO, and no difference was observed between the 2 groups (*p*>0.05). We further examined the vascular density in the MCA territory and found that the vessel density was increased in the rapamycin-treated rats compared to that in the MCAO-alone rats following MCAO (**Fig. 5A**). The diameters of the existing collaterals were all measured in the two main collaterals (**Fig. 5Bc**); the density and diameter of the collaterals in rapamycin-treated rats were increased compared to those of collaterals in MCAO-alone rats (**Fig. 5C**,* p*<0.05). This result demonstrated that rapamycin was beneficial for increasing vessel diameters in the existing collaterals and promoting angiogenesis.

**Figure 6:**
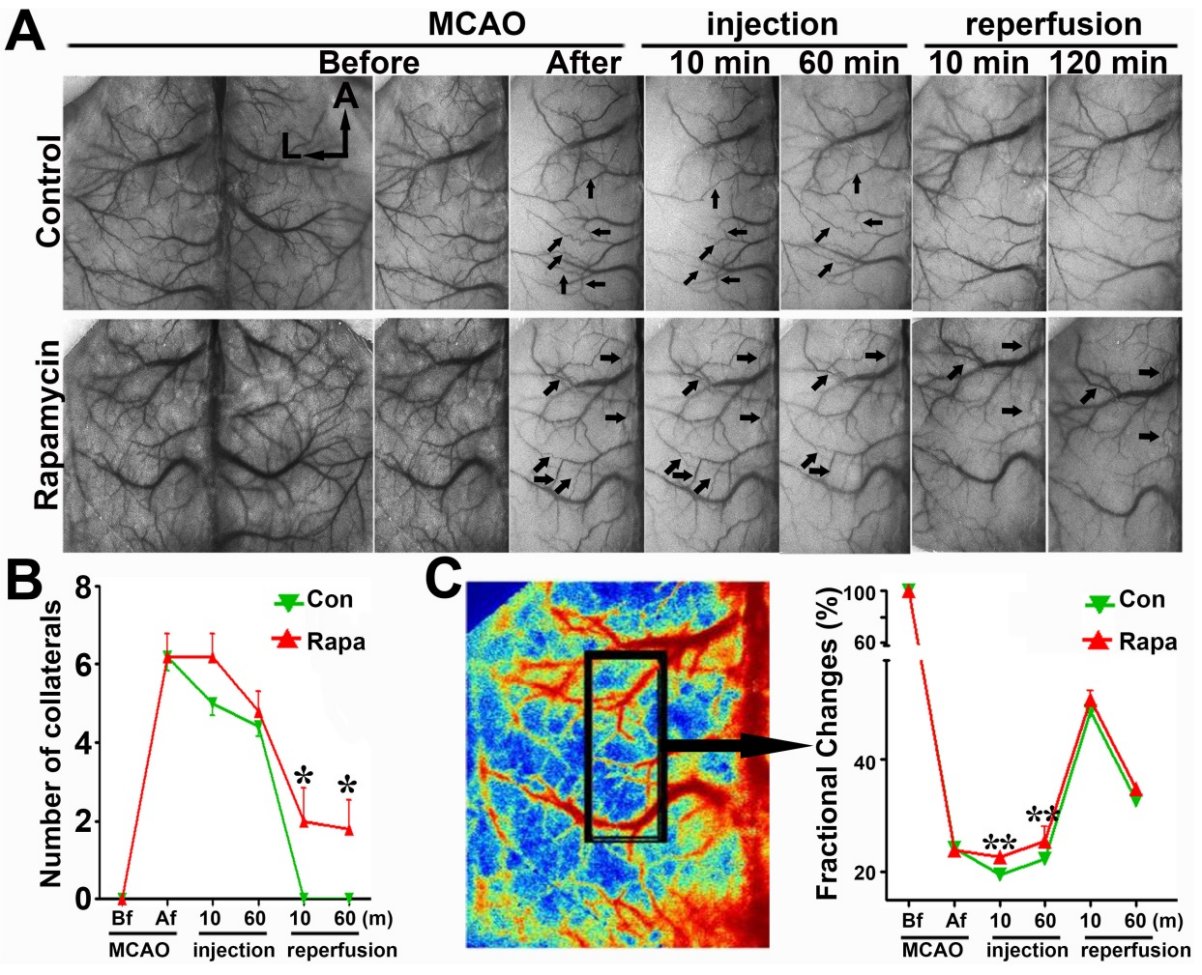
** LSCI shows that rapamycin improves the blood supply in the MCA territory. A)** Laser speckle imaging shows the effect of rapamycin on the promotion of cortical collateral circulation before and immediately after 10 and 60 min of MCAO and after 10 and 120 min of reperfusion. The arrows indicate newly formed collateral circulation after 10 and 60 min of reperfusion. The bar graph shows the collateral circulation **(B)** and surface blood flow **(C)** after MCAO in rapamycin-treated and MCAO-alone mice. n=6 per group, *p<0.05, **p<0.01, rapamycin-treated vs. MCAO-alone rats.

To verify the SRA data, we examined the number of collaterals and fractional changes in mice via LSCI measurement. The number of cortical collaterals (**Figs. 6A and 6B,** *p*<0.05) and the perfusion level (**Fig. 6C,** *p*<0.01) were increased in the rapamycin-treated mice compared to those in MCAO-alone mice, suggesting that rapamycin could promote collateral perfusion in the ischemic region.

## Discussion

To better understand collateral perfusion, collateral formation and collateral function, we combined several superior resolution and new imaging techniques, including SRA, micro-CT imaging, and LSCI. Using these multimodal imaging systems, we investigated collateral perfusion, formation, and spatial distribution following ischemic stroke in living rodents dynamically and in real time. We demonstrated the following results: 1) four collaterals (ICA to PPA, ICA to OphA, and OA to OphA) gradually formed after MCAO between the intra- and extra-arteries in the skull base as determined via SRA. These collaterals could exist for at least 28 days following MCAO. 2) Two main collaterals from the PCA to the MCA territory in the deep brain were critical to quickly supplying blood flow after MCAO. 3) Abundant small vessels and capillaries existed on the surface of the cortex between the PCA and MCA and the ACA and MCA. These collaterals increased after MCAO and could partially reduce the cortical ischemic injury. 4) Rapamycin reduced the infarct volume and improved neurological function recovery in MCAO mice compared with those in the controls, and 5) rapamycin greatly enlarged the diameter of preexisting collaterals and promoted angiogenesis. Altogether, we established a method for applying a multiple modality imaging system to study brain collateral perfusion and dynamically promote angiogenesis in living animals.

Collateral circulation is crucial during ischemic stroke in both humans and animals 10, 13. Although several techniques have provided insight into collateral flow in patients with ischemic stroke, an ideal imaging modality has not been established for the demonstration or accurate measurement of collateral circulation 44, especially in the widely used rodent cerebral ischemia model. We used SRA, micro-CT, and LSCI to study the microvessel changes in rodents because each method has its own advantages and disadvantages. Digital subtraction angiography (DSA) provides the best temporal and spatial resolution for the assessment of collateral vessels and has been considered a gold standard for collateral studies compared with other methods 45. However, the spatial resolution of DSA is larger than 200 μm 36, which is not sufficient to study collateral circulation in rodents. Although micro-CT provides three-dimensional imaging with a high resolution for tissue samples, it is not available for use in living animals because the scan time is too long (more than 6 h for one mouse brain). The view through a scan window is very limited, and this method cannot be used to scan whole mouse brain sections. In addition, micro-CT cannot assess blood flow or functional information 46. Magnetic resonance angiography (MRA) is another technique that showing potential diagnostic value for the detection of collateral circulation; it shows a better resolution in imaging and can detect changes in the cerebral vasculature and hemodynamics in small animals 47. However, its high sensitivity and reconstruction might restrict its ability to detect changes in collateral circulation in the deep brain, such as collaterals of the leptomeningeal artery. New emerging imaging techniques, such as vessel wall MRI, transcranial Doppler (TCD) ultrasonography, and 4D CT or MRA, have been shown to detect collaterals in humans after stroke 48. A study also showed that 4D MRA can quantify the degree of collateral circulation via the circle of Willis in patients with carotid artery steno-occlusive disease, which could be comparable to DSA 49. However, using these newer imaging techniques to estimate collateral circulation in small animals, such as rodents, may take time because the current MRA resolution should be improved. Because each imaging method has its own advantages and limitations, we herein developed a multiple modality approach that provides very useful tools for studying cerebral vasculature in different locations and a purposeful system for studying whole brain collateral circulation. We believe that SRA is one of the best approaches to dynamically detect collateral circulation in the skull base and deep brain in living rodents because this region cannot be accessed by other approaches. LSCI and TPLSM are suitable approaches for the study of cortical collateral circulation because they can detect changes in small vessel and capillary morphology and regional blood flow and velocity 50, 51.

As discussed in our manuscript, we chose to use both rats and mice because rats were appropriate for the SRA study and mice were appropriate for the LSCI and TPLSM study. When using mice to perform LSCI, we incised only the scalp and exposed the skull. In rats, we needed to grind the skull until the bone became very thin for imaging. This operation is relatively invasive and complicated. We believe that using mice for LSCI allowed the influence of external factors on the collateral circulation to be avoided as much as possible. In addition, the collaterals in rats could be only somewhat different between different rat strains and could even differ in the same strain deriving from different vendors 52, 53. Studies also showed that collaterals were different between mouse strains 54-56. However, it would still be interesting to analyze the similarities and/or differences of the rapamycin-induced response of collaterals in rats vs mice in a future study.

A previous study was performed to determine the effect of collateral blood flow in different cerebrovascular hierarchies on neuropathology after focal ischemic stroke 57. The researchers characterized the surface communication network and between the surface communication network and subsurface microcirculation network using two-photon microscopy. While using SRA, we detected not only changes in surface blood circulation and collateral formation but also collateral arteries in the skull base and the deeper brain after ischemia. However, it should be noted that the SRA used in the experiment produced 2D imaging, with which it is difficult to accurately quantify the total microvasculature volume. Developing a 3D SRA technique would provide more precise morphology information at both the micro and macro vasculature levels. Furthermore, two- photon microscopes reach only limited depth, provide µm level resolution, and require invasively opening the skull. We used multiple modalities, including synchrotron, micro-CT, laser speckle, and two- photon, to study collateral circulation during cerebral ischemia. Combining both the SRA and LSCI techniques allowed for the clear visualization of the surface and deeper collateral circulation in the brain without craniotomy.

Using a multimodal imaging system, we demonstrated that abundant collateral circulation could prevent or delay permanent neural damage and improve functional recovery. The spatial structure of the brain vascular system has three hierarchies: larger main supply of arteries in the skull base, terminal branches in the deep brain, and small vessels and capillaries on the surface of the brain. The extra- and intracollaterals could be immediately perfused or gradually formed in living rodents after ischemic stroke. The collateral perfusion rate in the deep brain between the PCA and MCA was approximately 75% in the MCAO rats. Furthermore, the diameter of these collateral circulation vessels was consistently maintained for at least 28 days after MCAO. These data suggest that collaterals both exist and can be newly formed during an ischemic situation in the brain. Therefore, sustaining or promoting blood flow in the perifocal region can attenuate neuronal death and increase injured neuron survival, thus providing great benefits for neuronal functional recovery after stroke. However, currently available measurements cannot easily distinguish collaterals between potential capillary and venular angiogenesis from neocollateral formation or collateral remodeling, which might include both phenomena. We therefore believe that these abundant small vessel and capillary anastomoses detected immediately after brain ischemia were collateral remodeling. Enhanced amounts of small vessels and capillaries detected at later time points were more likely due to both remodeling and angiogenesis.

Neuronal damage is not uniform after cerebral ischemia; the functions of neurons in the perifocal region may temporarily be ceased, but their structure remains intact. How long these neurons potentially survive depends on the timing of collateral perfusion, especially during the first few hours after ischemia. In addition, the infarct area could be expanded due to collateral failure, and promotion of collateral perfusion and establishment of a new blood supply system could be a potential therapeutic target to extend the time window for further treatment 58.

In this study, we tested the effects of rapamycin on the vasculature. It is known that endothelium- derived NO is the dominant effector via which EC signals are sent to vascular smooth muscle cells to regulate blood flow and vascular functions, including regulating vascular tone, transferring vascular signals, stimulating angiogenesis, and promoting vascular remodeling, platelet aggregation, and leukocyte- endothelial interactions 59, 60. Phosphorylated eNOS could reduce the ischemic area via increasing eNOS enzymatic activity and NO production *in vivo* 61. Recent studies have shown that rapamycin induces small cortical vessel dilation, restores CBF and increases vascular density dependent on increased NO synthase activity in a mouse model of AD 28. Our SRA and LSCI results also demonstrated that rapamycin could increase the cortical vessel density and enlarge the diameter of arteries in collaterals of the skull base and the deep brain. In addition, SRA data further revealed that the numbers of collaterals increased after rapamycin treatment, which subsequently attenuated neurobehavioral injury in MCAO mice. How rapamycin affects collaterals requires further investigation. Several reports demonstrated that rapamycin could dilate vessels 28, 62, 63.

In conclusion, we developed a multiple modality technology to dynamically study the collateral circulation in living animals after ischemic stroke and the collateral response after rapamycin treatment. We believe that multiple modality technology could provide a powerful tool for the diagnosis and treatment of ischemic stroke.

## Supplementary Material

Supplementary table.

## Abbreviations

ACA: anterior cerebral artery
BVD: brain vascular density
CBF: cerebral blood flow
ECA: external carotid artery
ICA: internal carotid artery
LSCI: laser speckle contrast imaging
MCA: middle cerebral artery
MCAO: middle cerebral artery occlusion
micro-CT: microcomputed tomography
mTOR: mammalian target of rapamycin
NO: nitric oxide
NSS: neurological severity score
OA: olfactory artery
OphA: ophthalmic artery
PCA: posterior cerebral artery
SR: synchrotron radiation
TPLSM: two-photon laser scanning microscopy
