# Supplementary material for: Rapamycin Increases Collateral Circulation in Rodent Brain after Focal Ischemia as detected by Multiple Modality Dynamic Imaging
Source: Theranostics. 2019 Jul 9;9(17):4923–34. doi: 10.7150/thno.32676 (PMC6691378; doi:10.7150/thno.32676)
Supplement: Supplementary file 1 — Supplementary table. [file thnov09p4923s1.pdf]

Supplemental table

Table 1: General body and blood parameters before and after MCAO

Parameter	Before MCAO	After MCAO	After reperfusion
Blood pressure (mmHg)	92±12	94±12	87±12
Heart rate (Beat/min）	400±38	418±3.28	398±43
Body temperature (°C)	37.0±0.3	37.1±0.5	36.8±0.5
Blood glucose (mg/dL)	192±23.0	198±25.2	-
Blood pH	7.45±0.03	7.46±0.06	7.46±0.03
Blood O^2 (mmHg)	49±4.0	50±3.3	51±3.6
Blood CO^2 (mmHg)	36.6±3.45	37.2±3.61	36.4±3.05
